# Keeping PPE barriers in COVID-19 wards while doing proper auscultation

**DOI:** 10.1186/s13756-020-00854-y

**Published:** 2020-12-09

**Authors:** Einat Seidel, Shahar Luski, Yaarit Ribak, Ahmed Nama, Takeshi Saraya, Toshiaki Nishiyama, Yonatan Oster, Ran Nir-Paz

**Affiliations:** 1 Faculty of Medicine, Hebrew University of Jerusalem, Jerusalem, Israel; 2 Department of Clinical Microbiology and Infectious Diseases, Hadassah Hebrew University Medical Center, Jerusalem, Israel; 3 Department of Allergy and Immunology, Hadassah Hebrew University Medical Center, Jerusalem, Israel; 4 Department of Emergency Medicine, Hadassah Hebrew University Medical Center, Jerusalem, Israel; 5 Department of Respiratory Medicine, Kyorin University School of Medicine, Tokyo, Japan; 6 CEO, Digital Global Systems, Inc., Tokyo, Japan

**Keywords:** Auscultation, Stethoscope, Physical examination, COVID-19, Personal protective equipment (PPE)

## Abstract

The emerging COVID-19 pandemic poses many difficulties to medical professionals. One of them is the need to use personal protective equipment (PPE) in order to protect themselves and their families, while not compromising their care. Physical examination is one of the cornerstones of medical assessment but parts of it are nearly impossible to do while wearing protective equipment. In this brief report we demonstrate a novel wireless stethoscope and its use for treating suspected and proven COVID-19 patients, as a representative to other infectious diseases.

## Background

The emerging Coronavirus disease 2019 (COVID-19) poses a serious risk for medical personnel infection, which may in turn cause nosocomial spread and endanger staff ability to provide patient care [1]. SARS-CoV-2, the causative agent of COVID-19, is transmitted mainly through droplets and contact [2]. The World Health Organization (WHO) recommends that medical staff use appropriate precautions, including personal protective equipment (PPE) and use of disposable or dedicated medical equipment; PPE used in high-risk settings include face mask or respirator, disposable gloves, face shield, and in many medical centers including our own, waterproof coveralls [3].

Physical examination is a cornerstone of medical assessment, and heart and lung auscultation is one of its inherent core skills [4]. A full physical examination is required when assessing newly admitted patients and is also required for ongoing assessment of the respiratory and cardiac condition of hospitalized patients in COVID-19 wards, for example to rapidly identify life threatening complications such as pneumothorax or non-endotracheal intubation.

However, stethoscope use causes breaches of PPE since placement of earbuds requires breaking coverall protection, and stethoscope removal and placing in COVID-19 wards while not in use can lead to contamination of staff. Recently, a commentary suggested that stethoscopes should be abandoned favoring ultrasound due to those infection/contamination risks [5].

When faced with this conundrum, we found that physicians in our hospital were routinely breaking infection control guidelines to provide the best care for their patients. Here, we describe our risk-minimizing approach in examining suspected and confirmed COVID-19 patients combining adequate infection control and proper stethoscope use.

## Methods

**Fig. 1:**
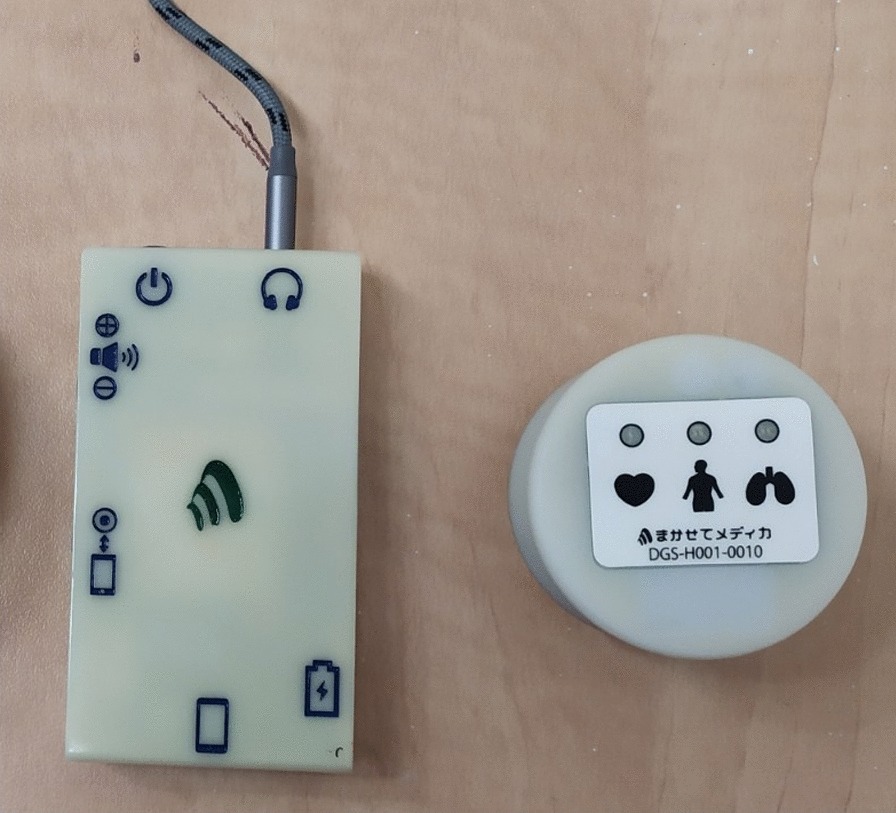
The wireless stethoscope (right), allowing different auscultation modes represented by the heart and lungs icons, the receiver unit (left) with different connections available for remote transmission of auscultation data, and the speaker cable (top) that allows the physician to hear heart and lung sounds without breaking PPE protocols

We employed a prototype wireless stethoscope system which allows the physician to auscultate patients without the need of earpiece insertion and PPE disruption, as an example to the concept of using an electronic stethoscope to maintain PPE protocols. The equipment, manufactured by Digital Global Systems (Japan), was originally developed for telemedicine and teaching purposes. It includes a wireless stethoscope-microphone with dedicated modes for high-pitched lung sounds or low-pitched heart sounds. The system is comprised of the stethoscope standalone head itself and a wireless receiver which can be connected either to a cell phone or to a headset. To facilitate physician acceptance and to minimize device interaction, we added a commercial portable waterproof speaker to allow safe auscultation without breaking PPE use protocols (Fig. 1). All parts can be disinfected between patients with 70% alcohol wipes. The system has a possibility of connecting a smartphone to the receiver unit to record and transmit findings, but to simplify system operation, we did not include it at this stage.

As a pilot, we introduced two devices, one to the Emergency Department area dedicated to suspected COVID-19 patients, and the second to a COVID-19 ward. Medical staff was instructed on operating and cleaning the unit.

Medical staff reactions observed in discussions with the authors, using narrative speech only, and were highly positive. The system provided audio quality superior to the dedicated disposable stethoscopes that were in use in the emergency department and in COVID-19 wards, and was easy to operate. Original sound samples can be heard in the Appendix, Patients’ consent was obtained before publication.

## Discussion

The COVID-19 outbreak is changing many longstanding practices, and medical staff must balance protecting themselves and providing good medical care [6]. Here, we present a technological approach that can help resolve one such conflict without compromising either best patient care or staff safety. This approach also promotes medical staff confidence in hospital protection protocols and enables them to use and apply medical skills in which they commonly practice; the use of similar device was demonstrated by Edelman and Weber several years ago [4] but is still not considered common practice. We have described the use of an electronic stethoscope in COVID-19 wards, to test this concept in its most practical environment; obviously the performance of this device and several similar devices should be compared and evaluated before recommending a specific product for routine use. Such electronic stethoscopes have multiple advantages beyond preserving PPE guidelines, including superior audio quality, the ability to transmit findings for consultation, and minimizing the number of medical staff exposed to patients. The fact that these devices are easily disinfected and can be transferred between patients permits the use of a small number of devices per ward. Obviously, the use of such devices may be applied to other infections control settings, facilitating better control of hospital acquired infections. In case of remote operation the physician should have an access to live video feed of the patient, as done in COVID-19 wards. The use of such technological solutions and continued search for approaches that mitigate the risks and difficulties of tackling the COVID-19 pandemic, while allowing healthcare professionals to practice the best standard of medicine, will facilitate better care for these patients. “Is there any thing whereof it may be said, See, this is new? It hath been already of old time, which was before us” [7].

## Supplementary Information

**Additional file 1.** **Sound 1** – sitting patient, mid lung: Coarse crackles are noted both in inspiratory and expiratory phase as well as squawk at inspiratory phase.

**Additional file 2.** **Sound 2** – sitting patient, base of the lung: late inspiratory crackles (fine crackles).

**Additional file 3.** **Sound 3** – Recovery phase - sitting patient, base of the lung: normal alveolar breathing.

## Data Availability

Not applicable.

## Abbreviations

COVID-19: Coronavirus disease 2019
WHO: World Health Organization
PPE: personal protective equipment
